# Genetic polymorphisms of toll-like receptors in leprosy patients from southern Brazil

**DOI:** 10.3389/fgene.2022.952219

**Published:** 2022-10-12

**Authors:** Priscila Saamara Masin, Hugo Alves Visentin, Laíse Nayana Sala Elpidio, Ana Maria Sell, Lorena Visentainer, Quirino Alves De Lima Neto, Joana Maira Valentini Zacarias, Patrícia Couceiro, Andressa Higa Shinzato, Manuel Santos Rosa, Paulo Rodrigues-Santos, Jeane Eliete Laguila Visentainer

**Affiliations:** ^1^ Immunology Institute, Faculty of Medicine, University of Coimbra, Coimbra, Portugal; ^2^ Immunogenetics Laboratory, Department of Basic Health Sciences, Post-Graduation Program in Biosciences and Phisiophatology, Maringá State University, Maringá, PR, Brazil; ^3^ Department of Medicine, Faculty of Medicine Science, Campinas State University, Campinas, SP, Brazil; ^4^ Immunology and Oncology Laboratory, Center for Neurosciences and Cell Biology (CNC), University of Coimbra, Coimbra, Portugal; ^5^ Center of Investigation in Environment, Genetics and Oncobiology (CIMAGO), Faculty of Medicine, University of Coimbra, Coimbra, Portugal; ^6^ Coimbra Institute for Clinical and Biomedical Research (iCBR), Faculty of Medicine, University of Coimbra, Coimbra, Portugal; ^7^ Center for Innovation in Biomedicine and Biotechnology (CIBB), University of Coimbra, Coimbra, Portugal; ^8^ Clinical Academic Centre of Coimbra (CACC), Coimbra, Portugal

**Keywords:** leprosy, toll-like receptors (TLR), TLR1, TLR2, innate immunity response

## Abstract

Leprosy is a chronic disease and also a global health issue, with a high number of new cases per year. Toll-like receptors can respond to mycobacterial molecules in the early stage of infection. As important components of the innate immune response, alterations in genes coding for these receptors may contribute to susceptibility/protection against diseases. In this context, we used a case-control study model (183 leprosy cases vs. 185 controls) to investigate whether leprosy patients and the control group, in southern Brazil, have different frequencies in *TLR1* (*TLR1* G>T; rs5743618), *TLR2* (*TLR2* T>C, rs1816702 and rs4696483), and *TLR4* (*TLR4* A>G, rs1927911) polymorphisms. Analysis of the *TLR1* 1805G>T polymorphism presented the *G/G* genotype more frequently in the control group. *TLR2* T>C rs1816702 and *TLR2* T>C rs4696483, the T/T and C/T genotype, respectively, were more frequent in the control group than in leprosy patients, suggesting protection from leprosy when the T allele is present (rs4696483). Haplotype analyses between *TLR1* (rs5743618) and *TLR2* (rs1816702 and rs4696483) polymorphisms suggest risk for the presence of the TCC haplotype and protection in the presence of the TCT haplotype. This study suggests that polymorphisms in *TLR1* and *TLR2* are factors that may contribute to development/resistance of leprosy.

## Introduction

Leprosy is an infectious disease caused by *Mycobacterium leprae* and *M. lepromatosis* (Han and Silva 2014). According to official reports, in 2019, there were 202,256 new leprosy cases registered from 161 countries, 14,893 of which were children below 14 years, and the new case detection rate among children was recorded at 7.9 children per million (World Health Organization, 2017) In 2018, the total number of leprosy new cases in Brazil was 22,940, of which 1,718 were in children under 15 years of age, corresponding to 7.5% and a detection rate of 3.72 cases per 100,000 inhabitants (Brasil, Ministério da Saúde, 2018). Despite these high numbers, leprosy is still classified as a stigmatizing and neglected disease (Yamey and Hotez 2007). The disease affects the skin, peripheral nerves, mucosa of the upper respiratory tract, and eyes. Leprosy is curable with multidrug therapy (MDT); however, if untreated, it can cause progressive and permanent damage to the skin, nerves, limbs, and eyes (World Health Organization, 2017).

In recent years, several genes have been associated with the development of leprosy, and the innate immune response pathways involved converge on the main hypothesis that genes are involved in susceptibility to the disease in two distinct steps: for leprosy per se and for the development of the different clinical forms, depending on the bacillary load in the host, which is influenced by genetic and external factors (Prevedello and Mira 2007). Genetic factors can participate in determining the immune response course in which cells and receptors will be activated to combat the infection. Twin studies (Chakravartti and Vogel, 1973), familial clustering (Shields et al., 1987), and segregation analyses (Abel et al., 1995) suggested that host genetics plays an important role in susceptibility to this infectious disease, with the heritability accounting for up to 57% of that of susceptibility, supporting an understanding of the immunity against *M. leprae* and providing insight into the host–pathogen relationship. Although several genetic *loci* have been associated with susceptibility to developing leprosy, a group conducted a large-scale study based on a case-control study with leprosy using a gene-centric 50-K microarray, covering variants in 2,092 genes throughout the genome (Keating et al., 2008) and found the *TLR1* and *HLA-DRB1/DQA1* genes as main determinants of leprosy susceptibility. They also observed a high degree of population differentiation at the *TLR1* gene, suggesting that mycobacterial diseases may have contributed to the evolution of this *locus* (Wong et al., 2010).

Toll-like receptors (TLRs) are pattern recognition receptors (PRRs) that play a crucial role in activation of innate immune response by detecting potential harmful molecules derived from pathogens. The PRRs are expressed predominantly in the host antigen-presenting cells (APCs) and recognize diverse pathogen-associated molecular patterns (PAMPs). Activation of TLRs by PAMPs leads to an upregulation of signaling pathways to modulate the host’s inflammatory response.

TLRs identified in humans are categorized by extracellular transmembrane (TLR1, TLR2, TLR4, TLR5, TLR6, and TLR11) and intracellular group (TLR3, TLR7, TLR8, and TLR9). TLRs on the extracellular transmembrane mainly recognize microbial membrane components such as lipids, lipoproteins, and proteins. TLR4 recognizes bacterial lipopolysaccharide (LPS). They can even form heterodimers to recognize its ligands, such as TLR2 along with TLR1 or with TLR6, which recognizes a wide variety of PAMPs, including lipoproteins, peptidoglycans, lipoteichoic acids, zymosan, mannan, and tGPI-mucin (Kawai and Akira 2011). TLR1/TLR2 heterodimers recognize mainly triacylated lipopeptides, whereas TLR2/TLR6 heterodimers recognize diacylated lipopeptides (Takeuchi et al., 2001). TLR1/2 heterodimers induce a different immune response against pathogens as compared to TLR2/6 heterodimers (Misch et al., 2008). When TLR1/2 molecules are absent, less induction of early cytokines was observed, whereas TLR2/6 could modulate the balance between a Th1/Th2 immune response.

In this context, alterations in their functions or inactivation of TLRs are mainly caused by mutations in the *TLR* gene that affect the normal functioning of these receptors. Based on the participation of Toll receptors in modulating the immune response to infection by *M. leprae*, we suggested that genetic variations in the *TLR1*, *TLR2*, and *TLR4* genes are somehow associated with leprosy.

## Materials and methods

### Study subjects

This case-control study was approved by the Human Research Ethics Committee at Maringa State University—Brazil (CEP no 464.158). Following written informed consent, a total volume of 10 ml of blood was obtained from all study participants. The studied populations were from the North and Northwest regions of the state of Paraná (22°29′30″-26°42′59″S and 48°02′24″-54°37′38″W), southern Brazil.

### DNA extraction

Genomic DNA samples from 183 patients and 185 controls were isolated from buffy-coat, using the Bio ™ DNA extraction kit (BiometrixDiagnóstica, Curitiba, PR, Brazil), following the manufacturer’s protocol. When necessary, the salting-out method with some modifications has also been used (John et al., 1991; Cardozo et al., 2009). The concentration and quality of the DNA were analyzed by optical density in a Thermo Scientific Nanodrop 2000^®^ apparatus (Thermo Fisher Scientific, Waltham, MA, United States).

### SNP analysis

Four SNPs were genotyped in *TLR* genes: *TLR1* 1805G>T (rs5743618), *TLR2* T>C (rs1816702), *TLR2* T>C (rs4696483), and *TLR4* A>G (rs1927911) by the PCR-SSP (polymerase chain reaction-sequence-specific primer) technique. The primers were designed and optimized at the Institute of Immunology, in the Faculty of Medicine–Coimbra University, Portugal (Table 1), and instructions were followed from TLR box 1.0tbox10, Instructions manual, Biocant (2010) (Biocant 2010). The amplification was standardized in a final volume of 15 μl: 150–300 nmol of each primer, 10 ng of DNA, 1x Standard Taq Reaction Buffer (New England Biolabs, United Kingdom), 4 mmol MgCl_2_, 3% glycerol, 0.15 mg of cresol red, 100 µmol of each dNTP, and 0.1 Unit of Taq DNA Polymerase (New England Biolabs, United Kingdom) in a thermocycler (Applied Biosystems, Foster City, CA, United States), using the following parameters: one cycle: 96°C, 1 min; five cycles: 96°C, 25 s, 70°C, 45 s, 72°C, 30 s; 21 cycles: 96°C, 25 s, 65°C, 45 s, 72°C, 30 s; four cycles: 96°C, 25 s, 55°C, 1 min, 72°C, 1 min; 72°C, 10 min; hold at 4°C (optional). Next, the amplified products were separated on 2% agarose gel electrophoresis and compared by molecular sizes, after gel staining with SYBR^®^ Safe (Life Technologies^®^, United States). Positive reactions were observed under UV light and photo-documented.

**TABLE 1:** Sequence of primers used for genotyping TLR1, TLR2, and TLR4 by PCR-SSP.

SNP	Forward primer	Forward sequence 5′–3′	Reverse primer	Reverse sequence 5′–3′	Specific band
rs5743618	TLR-1 602 FG	5′-TGT​GAC​CTC​CCT​CTG​CAG-3′	TLR-1 602 R	5′GAA​GGC​AAA​TCT​GCA​TAC​C3′	218bp
	TLR-1 602 FT	5′-CTG​TGA​CCT​CCC​TCT​GCA​T-3′	TLR-1 602 R	5′GAA​GGC​AAA​TCT​GCA​TAC​C3′	218bp
rs1816702	TLR-2 UTR 1FC	5′TTA​GAA​TTA​CAA​TGG​ACT​GCC3′	TLR-2 UTR 1R	5′GTG​TTC​CTA​AGC​CAC​AAC​AA3′	348bp
	TLR-2 UTR 1FT	5′CTT​AGA​ATT​ACA​ATG​GAC​TGC​T3′	TLR-2 UTR 1R	5′GTG​TTC​CTA​AGC​CAC​AAC​AA3′	348bp
rs4696483	TLR-2 UTR 2FC	5′CAA​ACA​ACC​AAA​CAA​AAA​TCG​C3′	TLR-2 UTR 2R	5′TGA​GGC​CAC​ATT​TAA​CAC​TA3′	396bp
	TLR-2 UTR 2FT	5′CAA​ACA​ACC​AAA​CAA​AAA​TCG​T3′	TLR-2 UTR 2R	5′TGA​GGC​CAC​ATT​TAA​CAC​TA3′	396bp
rs1927911	TLR-4 int 1F	5′TGT​GGT​TTA​GGG​CCA​AGT​C3′	TLR-4 int 1RC	5′AGG​GTC​AAT​GAG​CCA​AGG3′	298bp
	TLR-4 int 1F	5′TGT​GGT​TTA​GGG​CCA​AGT​C3′	TLR-4 int 1RT	5′AGG​GTC​AAT​GAG​CCA​AGA3′	298bp
Control	12 WS C3	GCA​TCT​TGC​TCT​GTG​CAG​AT	11 WS C5	TGC​CAA​GTG​GAG​CAC​CCA​A	790bp

### Statistical analysis

Statistical analysis was performed using SNPStats software (Sole et al., 2006) (https://www.snpstats.net/start.htm) and OpenEpi program Version 3.01 (https://www.openepi.com/Menu/OE_Menu.htm). The association of polymorphisms with the disease was evaluated using Chi-square and logistic regression analysis. Student’s t-test was used to compare the differences in age, and Fisher’s exact test was used to compare the differences in gender between groups. The association tests were performed for co-dominant, dominant, recessive, over-dominant, and log-additive genetic inheritance models, and the better inheritance model was chosen according to minor Akaike Information Criteria (AIC). The odds ratio with 95% confidence intervals was deemed only for significant *p*-values. All tests were carried out using a significance level of 5%. Genotype frequency distributions were evaluated to ensure Hardy–Weinberg equilibrium for all genes in the populations. To obtain an adequate minimum number of samples for carrying out this study with adequate statistical power (≥80%), the quantitative calculation software QUANTO (www.biostats.usc.edu/software) was used. For this purpose, we considered the less frequent allele (0.247 for *TLR2* rs1816702), population risk (0.1%), and OR of 2.0 (a medium-effect size). The linkage disequilibrium, haplotype block analysis, and haplotype population frequency estimation were performed using Haploview software (Barrett et al., 2004).

## Results

### Clinical characteristics

A total of 183 cases were enrolled in this study, in which 53.55% were male and 45.45% were female with a mean (SD) age of 50.85 (±14.34), and were primarily diagnosed by specialists in Dermatology at the Public Intermunicipal Health Consortium (CISAMUSEP) – 15th Regional Health Department of the State of Paraná. The control group was composed of 185 individuals: 59.46% male and 40.54% female, with a mean (SD) age of 51.30 (±16.0) years, healthy and non-related, and carefully matching to patients in relation to mean age, gender rates, and residence in the same geographical area. Due to the significant miscegenation of the Brazilian population, we considered patients and controls as a mixed ethnic group (Caucasian, Mulatto, and Black) according to Parra et al. (2003). The characteristics of patients and healthy subjects are shown in Table 2.

**TABLE 2:** Characteristics of leprosy patients and control group.

		Leprosy per se N = 183	Control, N = 185	*P*	OR	95% CI
Variable		n (%)	n (%)			
Age: mean ± SD		50.85 ± 14.34	51.30 ± 16.0	ns		
Age	≤40	38 (20.77)	62 (33.51)			
	>40	145 (79.23)	123 (66.49)	0.008	1.92	1.17–3.17
Gender	Male	98 (53.55)	110 (59.46)	ns		
	Female	85 (46.45)	75 (40.54)	ns		

n, number of individuals; N, population size; %: percentage; SD, standard deviation in years; *P*, *p-*value; ns, not significant; OR, odds ratio; CI, confidence interval.

### Allele and genotype distributions

The distribution of the genotype frequencies for all *TLR* genes was analyzed and was consistent with the Hardy–Weinberg equilibrium (*p* > 0.05). The distribution of the allele and genotype was also performed after adjustment to different parameters, like age, gender, and ethnic group, and we observed no significant association between these parameters, except age (Table 2).

*TLR1* G>T (rs5743618), *TLR2* T>C (rs1816702), *TLR2* T>C (rs4696483), and *TLR4* A>G (rs1927911) genotype and allele frequency distributions are summarized in Table 3, as well as the better inheritance model according to the minor AIC for each polymorphism. The association tests were performed for co-dominant, dominant, recessive, over-dominant, and log-additive genetic inheritance models, and complete analysis results are shown in Supplementary Table S1 in supporting information.

**TABLE 3:** *TLR1*G>T (rs5743618), *TLR2*T>C (rs1816702), *TLR2* T>C (rs4696483), and *TLR4* A>G (rs1927911) genotype and allele frequency distributions between leprosy patients and control group.

Genotype and allele model	Leprosy per se n (%)	Control n (%)	*P*	OR	CI
***TLR1* G>T (rs5743618)**	**N = 162**	**N = 181**			
Codominant					
T/T	57 (35.2)	64 (35.4)	*Ref*		
G/T	90 (55.6)	84 (46.4)	Ns		
G/G	15 (9.3)	33 (18.2)	Ns		
Recessive^a^					
T/T-G/T	147 (90.7)	148 (81.8)	*Ref*		
G/G	15 (9.3)	33 (18.2)	0.015	0.46	0.24–0.88
Allele					
T	204 (63.0)	212 (58.6)	*Ref*		
G	120 (37.0)	150 (41.4)	Ns		
***TLR2* T>C (rs1816702)**	**N = 117**	**N = 168**			
Codominant					
C/C	85 (72.7)	98 (58.3)	*Ref*		
C/T	29 (24.8)	57 (33.9)	Ns		
T/T	3 (2.6)	13 (7.7)	0.02	0.27	0.07–0.97
Log-additive^a^	—	—	0.0056	0.56	0.36–0.85
Allele					
C	199 (85.0)	253 (75.3)	*Ref*		
T	35 (15.0)	83 (24.7)	0.006	0.54	0.34–0.84
***TLR2* T>C (rs4696483)**	**N = 139**	**N = 164**			
Codominant^a^					
C/C	74 (53.2)	41 (25)			
C/T	55 (39.6)	79 (48.2)	<0.0001	0.39	0.23–0.64
T/T	10 (7.2)	44 (26.8)	<0.0001	0.13	0.06–0.28
Allele					
C	203 (73.0)	161 (49.1)	*Ref*		
T	75 (27.0)	167 (50.9)	<0.0001	0.36	0.25–0.51
***TLR4* A>G (rs1927911)**	**N = 147**	**N = 163**			
Codominant					
C/C	80 (54.4)	85 (52.1)	*Ref*		
C/T	48 (32.6)	65 (39.9)	ns		
T/T	19 (12.9)	13 (8)	ns		
Allele					
C	208 (70.8)	235 (72.1)	*Ref*		
T	86 (29.2)	91 (27.9)	ns		

a The better inheritance model according to minor Akaike Information Criteria. N, population size; n, number of individuals with the allele or genotype; %, allele and genotype frequencies x100; *P*, *p*-value; *Ref*, genotype used as the reference.

Analysis of the *TLR1* 1805G>T (rs5743618) polymorphism showed the *G/G* genotype to be more frequent in the control group (9.3% vs. 18.8%, *p* = 0.015) than in the recessive genetic inheritance model (*T/T-G/T* vs. *G/G*).

Significant differences were observed in *TLR2* polymorphisms (rs1816702 and rs4696483). For the *TLR2*T>C (rs1816702) polymorphism, the T/T genotype was less frequent in leprosy patients than in the control group (2.6% vs. 7.7%, *p* = 0.02), in the codominant genetic inheritance model, as well as the T allele (15.0% vs. 24.7%, *p* = 0.006). The protection against leprosy associated with the T allele was also observed in the log-additive genetic inheritance model (*p* = 0.0056, OR = 0.56), in which each copy of C modifies the risk in an additive form. Additionally, in *TLR2* T>C (rs4696483) polymorphism, the *T/T* and *C/T* genotypes were observed at a lower frequency in the leprosy patients than in the control group (7.2% vs. 26.8% and 39.6% vs. 48.2%, *p* < 0.0001), suggesting protection against leprosy disease when the T allele is present. For *TLR4* A>G (rs1927911) polymorphism, the frequencies found were proportionally distributed between patients and the control group.

### Haplotype analyses

Haplotype analyses were performed for the SNPs of *TLR1* (rs5743618) and *TLR2* (rs1816702 and rs4696483) genes. Significant linkage disequilibrium among SNPs was not observed. The haplotypes generated were compared between cases and controls (Table 4). We found eight haplotypes among *TLR1* and *TLR2* SNPs. Three of them showed frequencies with a *p*-value lower than 0.05 compared to the control and patients’ group. However, upon statistical corrections, only two of them retained their significant value: TCC (OR: 2.5; CI: 1.79–3.48), suggesting the risk to develop the disease, and TCT (OR: 0.55; CI: 0.37–0.81), suggesting protection (Table 4).

**TABLE 4:** Haplotype associations between TLR1 (rs5743618) and TLR2 (rs1816702 and rs4696483) polymorphism.

Haplotype association	F	Case, control ratios	*p*-value	OR	CI
TCC	0.294	137.2: 214:8, 75.3: 294.7	<0.000001	2.5	1.79–3.48
GCC	0.229	87.1: 264.9, 78.1: 291.9			
TCT	0.189	50.4: 301.6, 86.2: 283.8	0.0021	0.55	0.37–0.81
GCT	0.086	21.8: 330.2, 40.1: 329.9			
TTT	0.081	20.7: 331.3, 38.1: 331.9			
TTC	0.041	12.5: 339.5, 17.2: 352.8			
GTT	0.040	11.2: 340.8, 18.0: 352.0			
GTC	0.039	11.1: 340.9, 17.0: 353.0			

F, frequency percentage; *P*, *p-*value; ns: not significant; OR, odds ratio; CI, confidence interval.

## Discussion

In this study, we used a case-control study model in 183 leprosy cases and 185 controls to investigate whether leprosy patients and the control group in southern Brazil have different frequencies in *TLR1* (*TLR1* G>T; rs5743618), *TLR2* (*TLR2* T>C, rs1816702 and rs4696483) and *TLR4* (*TLR4* A>G, rs1927911) polymorphisms (Table 3). Due to the small number of samples, we decided not to include the analyses performed on the leprosy subtype groups.

As presented in our *Results* section and in Table 3, the frequency of *TLR1* allele G (rs5743618 - I602S) was highest in the control group as compared to the group of leprosy patients. *TLR1* rs5743618 (1805G>T, I602S) is a non-synonymous polymorphism that results in defective TLR1 trafficking to the cell membrane (Misch et al., 2008), resulting in the inability of TLR1 to participate in heterodimer formation with TLR2 and insufficient signaling in response to mycobacterial lipopeptides. Based on its function and functional importance, studies have been carried out and questions have been raised about the behavior of this polymorphism in infections, leprosy, and on how this polymorphism is distributed in different populations.

Our observations suggest that TLR1 1805G >T polymorphism showed the presence of the highest frequencies of the G allele in the control group in comparison to the patient group, and this SNP might be associated with resistance against leprosy development in the south Brazilian population. Misch et al. (2008) characterized a non-synonymous SNP, 1805G>T (I602S), in the transmembrane domain of *TLR1* that regulates signaling in response to synthetic TLR1 (Hawn et al., 2007) and found that the *TLR1* variant 1805G is associated with protection from reversal reaction (Johnson et al., 2007). They also reported that this polymorphism is associated with protection from leprosy in Turkey and that the *TLR1* signaling defect is caused by a complete absence of TLR1 on the surface of monocytes in GG individuals (Johnson et al., 2007). Hart and Tapping (2012) (Hart and Tapping 2012) suggested that the *TLR1* 602S variant protects against mycobacterial diseases. They demonstrated that monocytes and macrophages from 602S homozygous individuals were resistant to downregulation of MHC class II, CD64, and IFN responses when stimulated with a synthetic TLR1 agonist or mycobacterial membrane, compared to individuals 602I, suggesting that during evolution, mycobacteria have subverted the TLR system in ways that are advantageous to establishing and maintaining infection (Hart and Tapping 2012).

On the other hand, another group from Brazil investigated the same polymorphism in patients with malaria and found that the 602S variant was an associated risk factor for the development of this disease (Leoratti et al., 2008). Malaria is an infectious disease that also requires TLR1/2, TLR4, and TLR6 to form an immune response. However, this study was performed in the northern region of Brazil, covering a population from three areas of endemicity in the Amazonian region of Brazil with predominantly Amerindians, and a few individuals who were white or black also participated in the study. The *TLR1* (rs5743618) G allele was also associated with higher IFN-γ levels after BCG vaccination, and this allele was also associated with increased expression of T cell cytotoxicity molecules (Randhawa et al., 2011), supporting the immune response. This SNP was also investigated in Chinese (Zhang et al., 2011) and another Brazilian population (de Sales Marques et al., 2013); however, the authors did not find any significant differences.

The 1805G>T (I602S) polymorphism worldwide has a variable frequency in ethnic groups. In the Turkish population, the 602S variant has a frequency of 43% (Johnson at al. 2007), while among African Americans and Vietnamese individuals, it has a frequency of 25 and 1%, respectively (Hawn et al., 2007). Genetic association studies using different ethnic groups are vulnerable to positive associations in relation to the variant studied. This can be the result of the mixture of ethnicities in the population of interest, which can not only describe genuine genetic associations but also false-positive results. Previous studies demonstrated an association between polymorphisms in immune response and genomic ancestry (Crişan et al., 2011; Cassiano et al., 2015). Thus, in mixed populations, it is essential to study the haplotype frequencies and their associations with the levels of ancestry (Guimarães et al., 2018).

Studies show that the region of the *TLR1*, *TLR6*, and *TLR10* genes is under natural selection (Coop et al., 2009; Pickrell et al., 2009; Enard et al., 2010) and it has “signatures” of recent positive selection in Europeans (Barreiro et al., 2009; Laayouni et al., 2014). The Brazilian population has a major contribution of European ancestry (around 75%–77%) followed by African and Amerindian contributions, with the highest proportion of European ancestry in the south (87.7%) (Callegari-Jacques et al., 2003; Lins et al., 2010). Due to the importance of TLR receptors and their relevance in infectious diseases, Guimaraes et al*.* (2018) analyzed the connection between genomic ancestry of 24 polymorphisms distributed across five TLR genes (*TLR1*, *TLR2*, *TLR4*, *TLR6*, and *TLR9*) in a population from southeastern Brazil. They evaluated the influence of Brazilian population admixture on the distribution of these polymorphisms, and the results indicated that G allele prevalence in the *TLR1* gene increases in European ancestry and that this variant has evolved neutrally in Brazil and it has not been under positive selection since admixture (Guimarães et al., 2018). According to Leoratti et al. (2008), the “hypo-responsiveness” caused by the 602S variant could explain the high prevalence of asymptomatic malaria individuals in areas of southeastern Brazil (Leoratti et al., 2008). Although malaria and leprosy are diseases with different immune responses, the participation of toll-like receptors in triggering the (Sales. Marques et al., 2013) immune response leads us to reflect on the hypothesis that the same could be happening to those who spontaneously recover from leprosy. However, further studies must be carried out to support this hypothesis.

TLR2/TLR1 as heterodimers has an important role in immune response in infectious diseases. Several studies have shown the importance of TLR in the inflammatory process in the face of infection (Oliveira-Nascimento et al., 2012). TLR2 polymorphisms have been investigated in leprosy disease in different populations (Malhotra et al., 2005; Bochud et al., 2008) and have contributed to the TLR investigations in the innate immune response to leprosy. In our study, we found significant differences in *TLR2* polymorphisms rs1816702 and rs4696483. For the *TLR2* T>C (rs1816702), the T/T genotype was less frequent in leprosy patients than in the control group (2.6% vs. 7.7%) in the co-dominant genetic inheritance model, as well as the T allele (15.0% vs. 24.7%). The protection from leprosy associated with the T allele was also observed in the log-additive genetic inheritance model, where each copy of C modifies the risk in an additive form. Also, in the *TLR2* T>C (rs4696483) polymorphism, the *T/T* and *C/T* genotype had lower frequency in the leprosy patients than in the control group (7.2% vs. 26.8% and 39.6% vs. 48.2), suggesting protection from leprosy, whenever the T allele is present. To the best of our knowledge, this is the first study on leprosy and *TLR2* (rs1816702 and rs4696483) polymorphisms.

It is interesting to note that haplotype analysis of the three polymorphisms evaluated showed that in the presence of the rs5743618 polymorphism, the T allele (*TLR1* 602I) does not seem to interfere in defining the risk/protection to the development of leprosy since it is the allele change in the *TLR2* gene that shows a difference in the association with the disease, that is, when the T allele of the *TLR1* gene is present if the C allele of *TLR2* (rs4696483) is present, the haplotype formed suggests a risk of developing leprosy. Also, in the presence of the same *TLR1* T allele, when the allele present is the *TLR2* T allele, the data suggest protection against the development of leprosy, showing the predominance of the *TLR2* polymorphism in the presence of the defective *TLR1* allele. Thus, the allelic and haplotypic results are in agreement with those found in the literature and with the allele frequency found in the Brazilian population.

Regarding *TLR4* A>G (rs1927911) polymorphism, the frequencies found were proportionally distributed between patients and the control group, as shown in Table 3. Santana et al. (2017) observed high production of IL-1-β and IL-17 in individuals with the A allele (Santana et al., 2017). Interleukin 1-β (IL1-β) is essential for amplification of the T-cell’s specific immune response, and its levels tend to decrease after multidrug therapy (Moubasher et al., 1998). This cytokine is also produced in high concentrations in multibacillary patients (Madan et al., 2011). Some SNPs in the *TLR4* gene have been the subject of research due to their importance in the recognition and targeting of various diseases, such as leprosy (Bochud et al., 2009; Suryadevara et al., 2013). Bochud et al. (2009) investigated the *TLR4* gene (896GA, 1196CT) in an African population and showed a protective effect of 896GA and 1196 TT against the development of leprosy (Bochud et al., 2009). A meta-analysis study showed an association of *TLR4* polymorphisms with the risk of developing various infections, including Gram-positive bacteria, Gram-negative bacteria, and parasitic infections (Ziakas et al., 2013).

Genetic variants in *TLR* genes may contribute to different response phenotypes, including susceptibility to infection. TLR1 and TLR2 molecules are able to form heterodimers and recognize dead *M. leprae* cells, as well as TLR4, which can recognize LPS ligands. From the recognition of the antigen by the TLR receptor, intracellular signaling is initiated, highlighting a possible involvement of TLR in the immune response, affecting the clinical manifestations of leprosy. In this way, genetic variants in *TLR* genes may contribute to different response phenotypes, including susceptibility/resistance to leprosy.

## Conclusion

Our findings reinforce the idea of the participation of *TLR1 and TLR2* polymorphisms in the immune response to leprosy. Based on our findings, and in accordance with previous studies, we point to the *TLR1* (rs5743618) polymorphism as an interesting SNP to be further investigated in its possible role in modulating the immune response in leprosy.

## Data Availability

The original contributions presented in the study are included in the article/Supplementary Material; further inquiries can be directed to the corresponding author.
