# Supplementary material for: Genetic polymorphisms of toll-like receptors in leprosy patients from southern Brazil
Source: Front Genet. 2022 Oct 12;13:952219. doi: 10.3389/fgene.2022.952219 (PMC9596761; doi:10.3389/fgene.2022.952219)
Supplement: Supplementary file 1 [file Table1.DOCX]

**[Immunogenetics](https://www.springer.com/journal/251/)**

**Genetic polymorphisms of Toll -like receptors in leprosy patients from Southern Brazil**

Priscila S. Masin^1,2^, Hugo V. Alves ^2^, Laíse N. S. Elpidio^2^, Ana M. Sell^2^, Lorena Visentainer^3^, Quirino A. Lima Neto^2^, Joana M. V. Zacarias^2^, Patrícia Couceiro^4,5,6,7,8^, Manuel Santos-Rosa^1,5,6,7,8^, Paulo Rodrigues-Santos^1,4,5,6,7,8#^ and Jeane E. L. Visentainer^2#^

^1^Immunology Institute, Faculty of Medicine, University of Coimbra, Coimbra, Portugal.

^2^Immunogenetics Laboratory, Department of Basic Health Sciences, Maringá State University, Maringá, PR, Brazil.

^3^Department of Medicine, Faculty of Medicine Science, Campinas State University, Campinas, SP, Brazil.

^4^Immunology and Oncology Laboratory, Center for Neurosciences and Cell Biology (CNC), University of Coimbra, Coimbra, Portugal.

^5^Center of Investigation in Environment, Genetics and Oncobiology (CIMAGO), Faculty of Medicine, University of Coimbra, Coimbra, Portugal.

^6^Coimbra Institute for Clinical and Biomedical Research (iCBR), Faculty of Medicine, University of Coimbra, Coimbra, Portugal.

^7^Center for Innovation in Biomedicine and Biotechnology (CIBB), University of Coimbra, Coimbra, Portugal.

^8^Clinical Academic Centre of Coimbra (CACC), Coimbra, Portugal.

#PRS and JELV contributed equally and should be considered as senior authors.

Correspondence:

Jeane E. L. Visentainer (jelvisentainer@uem.br)

[**Immunogenetics**](https://www.springer.com/journal/251/)

**Supplementary 1: Genotype and allele frequency distributions for *TLR1* G>T (rs5743618), *TLR2* T>C (rs1816702), *TLR2* T>C (rs4696483), and *TLR4* A>G (rs1927911) polymorphisms between Leprosy *per se* patients and controls in codominant, dominant, recessive, over dominant and log-additive genetic inheritance models.**

|  | **Leprosy *per se*** | **Controls** |  |  |  |  |
| --- | --- | --- | --- | --- | --- | --- |
| **Genotypes and Alleles** | **n (%)** | **n (%)** |  |  |  |  |
| **Model** |  |  | ***P*** | **OR** | **CI** | **AIC** |
| ***TLR1* G>T (rs5743618)** | **N=162** | **N=181** |  |  |  |  |
| Codominant |  |  |  |  |  |  |
| T/T |  | 64 (35.4) | 0.039 | 1.00 |  | 474.0 |
| G/T | 90 (55.6) | 84 (46.4) |  | 1.20 | 0.76-1.91 |  |
| G/G | 15 (9.3) | 33 (18.2) |  | 0.51 | 0.25-1.04 |  |
| Dominant |  |  |  |  |  |  |
| T/T | 57 (35.2) | 64 (35.4) | 0.97 | 1.00 |  | 478.4 |
| G/T-G/G | 105 (64.8) | 117 (64.6) |  | 1.01 | 0.65-1.57 |  |
| Recessive |  |  |  |  |  |  |
| T/T-G/T | 147 (90.7) | 148 (81.8) | 0.015 | 1.00 |  | 472.6 |
| G/G | 15 (9.3) | 33 (18.2) |  | 0.46 | 0.24-0.88 |  |
| Overdominant |  |  |  |  |  |  |
| T/T-G/G | 72 (44.4) | 97 (53.6) |  |  |  | 475.6 |
| G/T | 90 (55.6) | 84 (46.4) |  |  |  |  |
| Log-additive | --- | --- | 0.22 | 0.82 | 0.60-1.13 | 477.0 |
| ***TLR2* T>C (rs1816702)** | **N=117** | **N=168** |  |  |  |  |
| Codominant |  |  |  |  |  |  |
| C/C | 85 (72.7) | 98 (58.3) | 0.02 | 1.00 |  | 384.1 |
| C/T | 29 (24.8) | 57 (33.9) |  | 0.59 | 0.34-1.00 |  |
| T/T | 3 (2.6) | 13 (7.7) |  | 0.27 | 0.07-0.97 |  |
| Dominant |  |  |  |  |  |  |
| C/C | 85 (72.7) | 98 (58.3) | 0.012 | 1.00 |  | 383.7 |
| C/T-T/T | 32 (27.4) | 70 (41.7) |  | 0.53 | 0.32-0.88 |  |
| Recessive |  |  |  |  |  |  |
| C/C-C/T | 114 (97.4) | 155 (92.3) | 0.05 | 1.00 |  | 386.1 |
| T/T | 3 (2.6) | 13 (7.7) |  | 0.31 | 0.09-1.13 |  |
| Overdominant |  |  |  |  |  |  |
| C/C-T/T | 88 (75.2) | 111 (66.1) |  | 1.00 |  | 387.1 |
| C/T | 29 (24.8) | 57 (33.9) | 0.096 | 0.64 | 0.38-1.09 |  |
| Log-additive | --- | --- | 0.0056 | 0.56 | 0.36-0.85 | 382.3 |
| ***TLR2* T>C (rs4696483)** | **N=139** | **N=164** |  |  |  |  |
| Codominant |  |  |  |  |  |  |
| C/C | 74 (53.2) | 41 (25) |  | 1.00 |  | 389.0 |
| C/T | 55 (39.6) | 79 (48.2) | <0.0001 | 0.39 | 0.23-0.64 |  |
| T/T | 10 (7.2) | 44 (26.8) | <0.0001 | 0.13 | 0.06-0.28 |  |
| Dominant |  |  |  |  |  |  |
| C/C | 74 (53.2) | 41 (25) | <0.0001 | 1.00 |  | 396.3 |
| C/T-T/T | 65 (46.8) | 123 (75) |  | 0.29 | 0.18-0.48 |  |
| Recessive |  |  |  |  |  |  |
| C/C-C/T | 129 (92.8) | 120 (73.2) | <0.0001 | 1.00 |  | 400.6 |
| T/T | 10 (7.2) | 44 (26.8) |  | 0.21 | 0.10-0.44 |  |
| Overdominant |  |  |  |  |  |  |
| C/C-T/T | 84 (60.4) | 85 (51.8) | 0.13 | 1.00 |  | 419.7 |
| C/T | 55 (39.6) | 79 (48.2) |  | 0.70 | 0.45-1.11 |  |
| Log-additive |  |  | <0.0001 | 0.36 | 0.25-0.52 | 387.1 |
| ***TLR4* A>G (rs1927911)** | **N=147** | **N=163** |  |  |  |  |
| Codominant |  |  |  |  |  |  |
| C/C | 80 (54.4) | 85 (52.1) | 0.22 | 1.00 |  | 431.9 |
| C/T | 48 (32.6) | 65 (39.9) |  | 0.78 | 0.48-1.27 |  |
| T/T | 19 (12.9) | 13 (8) |  | 1.55 | 0.72-3.35 |  |
| Dominant |  |  |  |  |  |  |
| C/C | 80 (54.4) | 85 (52.1) | 0.69 | 1.00 |  | 432.8 |
| C/T-T/T | 67 (45.6) | 78 (47.9) |  | 0.91 | 0.58-1.43 |  |
| Recessive |  |  |  |  |  |  |
| C/C-C/T | 128 (87.1) | 150 (92) | 0.15 | 1.00 |  | 430.9 |
| T/T | 19 (12.9) | 13 (8) |  | 1.71 | 0.81-3.60 |  |
| Overdominant |  |  |  |  |  |  |
| C/C-T/T | 99 (67.3) | 98 (60.1) | 0.19 | 1.00 |  | 431.2 |
| C/T | 48 (32.6) | 65 (39.9) |  | 0.73 | 0.46-1.16 |  |
| Log-additive | --- | --- | 0.73 | 1.06 | 0.76-1.48 | 432.8 |

N: population size; n: number of individuals with the genotype; %: genotype frequencies x100. *P*: *P*-value. AIC: Akaike Information Criteria.
